# Association of Metastatic Lymph Node Count and Primary Tumor Size with Disease-Free Survival in Stage II–III Gastric Cancer: A Retrospective Cohort Study

**DOI:** 10.3390/medicina62081528

**Published:** 2026-08-08

**Authors:** Emine Kanatsız, Yasin Sezgin, Yonca Yılmaz Ürün

**Affiliations:** 1 Department of Gastroenterology and Hepatology, Ümraniye Training and Research Hospital, İstanbul 34764, Turkey; 2 Department of Medical Oncology, Medical Faculty, Van Yüzüncü Yıl University, Van 65080, Turkey; dr.yasin1982@hotmail.com; 3 Department of Gastroenterology and Hepatology, Medical Faculty, Van Yüzüncü Yıl University, Van 65080, Turkey; dryoncayilmazurun@gmail.com

**Keywords:** gastric cancer, disease-free survival, lymph node ratio, log odds of positive lymph nodes, tumor size

## Abstract

*Background and Objectives:* The relative prognostic information provided by different nodal metrics and the independent role of primary tumor size remain unclear in gastric cancer. This study evaluated the association of metastatic lymph node count and primary tumor size with disease-free survival (DFS) in stage II–III gastric cancer (GC) treated with a surgery-first approach. *Materials and Methods:* This retrospective single-center study included 128 patients who underwent curative-intent gastrectomy between 2003 and 2019 without neoadjuvant treatment. A multivariable Cox model with clinically prespecified covariates was fitted. Metastatic lymph node count, pathological N (pN) category, lymph node ratio (LNR), and log odds of positive lymph nodes (LODDS) were each added separately to the same base clinical model and compared using the Akaike information criterion (AIC). *Results:* During a median follow-up of 114.3 months, 86 DFS events occurred. Each additional metastatic lymph node was independently associated with a higher hazard of a DFS event (HR, 1.049; 95% CI, 1.022–1.076; *p* < 0.001), and this association persisted across four sensitivity analyses. Primary tumor size was not independently associated with DFS. All four nodal metrics improved model fit, with LNR yielding the lowest AIC. *Conclusions:* Metastatic lymph node count was independently and robustly associated with DFS, whereas primary tumor size was not. Although LNR provided the best relative model fit, these within-cohort comparisons do not establish clinical superiority, and external validation is required.

## 1. Introduction

Gastric cancer (GC) is a major global health burden, accounting for an estimated 980,286 new cases and 641,554 deaths in 2024, making it the fifth most commonly diagnosed cancer and the fifth leading cause of cancer death worldwide [1]. For patients with resectable, locally advanced GC, multimodal treatment strategies combining radical gastrectomy with perioperative or adjuvant systemic therapy have improved clinical outcomes [2,3]. Current clinical guidelines likewise recommend a multidisciplinary, stage-adapted treatment strategy that takes into account tumor stage, resectability, and the patient’s performance status [3]. Nevertheless, a substantial proportion of patients undergoing curative-intent resection develop postoperative recurrence, and long-term survival outcomes remain suboptimal [4]. Given the substantial biological and clinical heterogeneity of GC [5], refinement of postoperative prognostic assessment remains important for identifying patients at higher risk of recurrence and informing surveillance and treatment strategies.

Numerous demographic, clinicopathological, inflammatory, nutritional, and treatment-related factors have been investigated as potential prognostic markers in patients undergoing curative-intent gastrectomy [6]. Among conventional clinicopathological factors, the extent of regional lymph node involvement is of particular prognostic importance [7]. The pathological N (pN) category is based on the absolute number of metastatic regional lymph nodes and represents one of the fundamental components of the tumor–node–metastasis (TNM) staging system [7]. However, an assessment based solely on the metastatic lymph node count may be influenced by the extent of lymphadenectomy, the total number of lymph nodes retrieved and pathologically examined, the methods of pathological evaluation, and stage migration.

Alternative nodal measures, including the lymph node ratio (LNR) and the log odds of positive lymph nodes (LODDS), have been proposed to partly mitigate these limitations of lymph node assessment [8]. LNR represents the proportion of retrieved lymph nodes that are metastatic [9,10], whereas LODDS represents the relationship between positive and negative lymph nodes on a logarithmic scale [11]. Higher LNR values have been associated with poorer recurrence-free survival and overall survival (OS) in patients with pathological stage II–III GC [10], and LNR has been identified as an independent risk factor for early recurrence in patients older than 65 years [9]. Similar prognostic associations have been demonstrated in other surgical patient populations [12,13,14]. In addition, pathological T (pT) and pN categories have been reported to be among the principal determinants of disease-free survival (DFS) and OS in stage I–III GC [15]. Nevertheless, few studies have directly compared the metastatic lymph node count, the pN category, LNR, and LODDS within a single statistical framework adjusted for the same clinical covariates. Consequently, the relative prognostic information provided by these nodal metrics has not yet been fully clarified.

Primary tumor size has also been investigated as a potential prognostic factor in GC. Although several studies have shown that larger tumor size is associated with poorer clinical outcomes, the proposed cut-off values and their prognostic implications vary considerably. Tsutsuyama et al. reported that a tumor size of 5 cm or greater was an adverse prognostic factor in patients with stage II–III GC receiving postoperative S-1 monotherapy [16]. In node-negative disease, OS has been shown to decline progressively with increasing tumor size [17]. Del Rio et al., by contrast, arrived at a substantially lower cut-off of 2.5 cm in a retrospective surgical cohort, with tumors below this threshold showing better OS [18]. Stage-stratified analyses have indicated that the prognostic effect of tumor size depends on depth of invasion: tumor size retained independent prognostic value in T2–T4 tumors, whereas no such association was observed in T1 tumors [19]. These findings suggest that the prognostic contribution of tumor size may vary according to pT category, nodal status, disease stage, treatment strategy, patient selection, and the covariates entered into the statistical model. It therefore remains unclear whether primary tumor size retains independent prognostic value in patients with pathological stage II–III disease when nodal disease burden and other key clinicopathological variables are accounted for simultaneously.

The aim of this study was to evaluate the association of the metastatic lymph node count and primary tumor size with DFS in patients with pathological stage II–III GC treated with a surgery-first approach. In addition, the metastatic lymph node count, the pN category, LNR, and LODDS were each entered separately into the same baseline clinical adjustment model to compare the prognostic information provided by these nodal metrics and the relative fit of the resulting models. Several sensitivity analyses were performed to assess the robustness of the association between the metastatic lymph node count and DFS.

## 2. Materials and Methods

### 2.1. Study Design and Patient Population

This retrospective, single-center cohort study was conducted at Van Yüzüncü Yıl University Faculty of Medicine, screening institutional medical records from January 2001 to January 2024. With the database closed in January 2024, the final eligible surgery-first cohort comprised patients who underwent gastrectomy between March 2003 and October 2019.

Patients were included if they were 18 years of age or older, had undergone gastrectomy with curative intent without neoadjuvant treatment, had no evidence of distant metastatic disease at surgery, and had a postoperative pathological stage of II or III. Curative-intent gastrectomy was defined as gastric resection undertaken to achieve macroscopically complete removal of the tumor in the absence of distant metastatic disease. Patients were retained in the cohort regardless of whether the surgical margins were microscopically negative (R0) or positive (R1), as long as the procedure was initially performed with curative intent. Patients undergoing palliative surgery, procedures without resection, or any other operation not regarded as curative were excluded. Patients were also excluded if they had multiple primary malignancies, pathological stage I or IV disease, or lacked sufficient clinical or pathological data for the planned analyses. Patients who had received neoadjuvant treatment were likewise excluded, in order to obtain a clinically more homogeneous surgery-first cohort and to avoid treatment-related changes in pathological tumor size, pT category, and lymph node involvement.

Treatment decisions were made by a multidisciplinary tumor board according to clinical staging, resectability, patient performance status, and the institutional treatment practices applicable during the corresponding period. Neoadjuvant or perioperative chemotherapy was not routinely used in this patient group at our institution during the surgical inclusion period; management therefore reflected the local standard of care at the time.

### 2.2. Data Collection and Clinicopathological Definitions

The recorded variables included the following: age, sex, comorbidities, smoking history, calendar period of surgery, surgical margin status, type of gastrectomy, extent of lymph node dissection, Lauren classification, tumor location, histological subtype, histological grade, pT and pN categories, pathological stage, perineural invasion, vascular invasion, serosal involvement, human epidermal growth factor receptor 2 (HER2) status, Eastern Cooperative Oncology Group (ECOG) performance status, receipt and type of adjuvant chemotherapy, number of chemotherapy cycles, receipt of adjuvant chemoradiotherapy, primary tumor size, total number of retrieved lymph nodes, and number of metastatic lymph nodes. Smoking history was retrospectively recorded as a binary variable (yes or no) based on the available medical records. Information on current vs. former smoking status, smoking intensity and duration, cumulative tobacco exposure, and smoking cessation was not consistently documented across the cohort.

All tumors were restaged retrospectively according to the eighth edition of the American Joint Committee on Cancer (AJCC)/Union for International Cancer Control (UICC) pathological TNM classification, based on the information recorded in the original surgical pathology reports [7]. The pT category was based on the depth of tumor invasion described in the pathology report, whereas the pN category was based on the recorded number of metastatic regional lymph nodes. Patients whose archival pathology reports lacked the information required for reliable restaging were excluded under the insufficient-data criterion.

Primary tumor size was defined as the maximum tumor diameter stated in the surgical pathology report and was analyzed as a continuous variable in centimeters. Surgical margins were classified as microscopically negative (R0) or microscopically positive (R1). The total number of lymph nodes removed from the surgical specimen and examined pathologically was termed the total retrieved lymph node count. The absolute number of histologically confirmed metastatic regional lymph nodes was termed the metastatic lymph node count.

The LNR was calculated by dividing the metastatic lymph node count by the total retrieved lymph node count. The LODDS was calculated as ln [(metastatic lymph nodes + 0.5)/(negative lymph nodes + 0.5)]. Negative lymph nodes were defined as the total number of retrieved lymph nodes minus the number of metastatic lymph nodes. The correction of 0.5 was added to allow calculation in patients with zero metastatic or zero negative lymph nodes.

To account for changes in patient selection, surgical practice, pathological assessment, adjuvant treatment, and surveillance over the long inclusion period, patient surgeries were categorized into two calendar periods: before 2012 and 2012 or later. The year 2012 was selected for analytical purposes because it approximated the median calendar year of surgery and yielded two nearly balanced groups (63 and 65 patients, respectively), rather than representing a specific change in treatment policy or clinical guidelines. Adjuvant chemotherapy regimens were classified as fluorouracil plus folinic acid or capecitabine (FUFA/capecitabine); capecitabine plus oxaliplatin (CAPOX); docetaxel, cisplatin, and fluorouracil (DCF); fluorouracil, leucovorin, and oxaliplatin (FOLFOX); or other regimens.

### 2.3. Outcome Definition and Follow-Up

DFS was the sole study endpoint and was defined as the interval from the date of surgery to the first documented locoregional, peritoneal, or distant recurrence, or death from any cause, whichever occurred first. Patients who died without previously documented recurrence were recorded as having a DFS event on the date of death. Patients who died after developing recurrence were counted as having a DFS event only at the date of first recurrence. Patients who were alive and free of recurrence were censored at the date of the last disease assessment. The causes of death in patients who died without documented recurrence could not be reliably ascertained from the available retrospective records; a cause-of-death sensitivity analysis was therefore not feasible.

Recurrence was defined as the development of locoregional recurrence, peritoneal dissemination, or distant metastatic disease during postoperative follow-up. Recurrence was established primarily by radiological imaging and was supported by histopathological confirmation when clinically indicated and available. The date on which recurrence was first clinically or radiologically documented was used as the event date. The anatomical pattern of the first documented recurrence was classified as locoregional, peritoneal, hepatic, pulmonary, bone, distant lymph node, ovarian, bladder, or synchronous multisite recurrence. Involvement of more than one anatomical site at the same assessment was classified as synchronous multisite recurrence. Percentages of recurrence patterns were calculated using only patients with documented recurrence as the denominator.

Postoperative follow-up was generally performed every 3 months during the first 2 years, every 6 months during years 3–5, and annually thereafter. Follow-up assessments included physical examination, laboratory testing, and radiological imaging as clinically indicated. Follow-up data were evaluated up to January 2024. Given the long study period, the exact timing of individual follow-up visits and the imaging modalities used could vary according to the treatment period and the clinical circumstances of each patient.

### 2.4. Statistical Analysis

Statistical analyses were performed using IBM SPSS Statistics for Windows, version 25.0 (IBM Corp., Armonk, NY, USA). Categorical variables are presented as frequencies and percentages, whereas continuous variables are summarized as medians with minimum and maximum values. The median follow-up time was estimated using the reverse Kaplan–Meier method. Follow-up time was likewise estimated by surgery period using the reverse Kaplan–Meier method, and the two periods were compared using the log-rank test. No statistical imputation was performed for missing data. Data on the covariates included in the primary multivariable survival model were complete for all 128 patients. All statistical tests were two-sided, and *p* values < 0.05 were considered statistically significant.

Clinicopathological characteristics were compared between patients treated before 2012 and those treated in 2012 or later. Categorical variables were compared using Pearson’s chi-square test or the two-sided Fisher’s exact test when expected cell counts were small. Continuous variables were compared using the Mann–Whitney U test.

Univariable Cox proportional hazards regression analyses were performed to evaluate the association of each demographic, clinicopathological, and treatment-related variable with DFS. Statistical significance in the univariable analyses was not used as a criterion for variable selection in the multivariable model.

The primary multivariable Cox proportional hazards model was specified a priori on clinical grounds and was fitted using the Enter method rather than an automated or stepwise variable selection procedure. The model included age, smoking history, surgery period, surgical margin status, pT category, ECOG performance status, receipt of adjuvant chemotherapy, primary tumor size, and metastatic lymph node count.

Age, primary tumor size, and metastatic lymph node count were modeled as continuous variables, with effects expressed per 1-year, 1 cm, and 1-node increase, respectively. Smoking history was compared as present vs. absent, surgery period as 2012 or later vs. before 2012, surgical margin status as R1 vs. R0, and adjuvant chemotherapy as administered vs. not administered. The reference categories were pT2 and ECOG performance status 0. Because two indicator parameters were used for each of pT category and ECOG performance status, the primary model included a total of 11 estimated parameters and was based on 86 DFS events. Associations were reported as hazard ratios (HRs) with 95% confidence intervals (CIs).

The linearity of the association between metastatic lymph node count and the log hazard was assessed by adding a Box–Tidwell transformation term, calculated as (metastatic lymph node count + 1) × ln (metastatic lymph node count + 1), to the fully adjusted Cox model, where 1 was added to permit the transformation in patients with no metastatic lymph nodes. Models with and without the transformation term were compared using the likelihood-ratio test.

Metastatic lymph node count, pN category, LNR, LODDS, pathological stage, and the dichotomized metastatic lymph node variable were not entered simultaneously into the same multivariable model, because they represent overlapping measures of nodal disease burden. To compare the alternative nodal metrics within a common adjustment framework, a base clinical Cox model was first fitted, including age, smoking history, surgery period, surgical margin status, pT category, ECOG performance status, receipt of adjuvant chemotherapy, and primary tumor size. Metastatic lymph node count, pN category, LNR, and LODDS were then added separately to this base model.

Nested models were compared using likelihood-ratio tests. Relative model fit was summarized using the −2 log likelihood and the Akaike information criterion (AIC). Lower AIC values were interpreted solely as indicating better relative fit within the present cohort; they were not taken as evidence of external validity, clinical utility, or superiority of one nodal metric over another. Because each nodal metric was evaluated in a separate model, the metrics were not mutually adjusted for one another.

The proportional hazards assumption of the primary multivariable Cox regression model was assessed by adding interaction terms between each covariate and the natural logarithm of analysis time. Global interaction tests were used for variables with more than two categories. Because a possible time-dependent effect was observed for pT4, an additional Cox model stratified by pT category was fitted as a sensitivity analysis. In this model, separate baseline hazard functions were allowed for pT2, pT3, and pT4 disease.

Additional sensitivity analyses were performed by restricting the cohort to patients with R0 resection, by additionally including the total retrieved lymph node count in the primary model, and by administratively censoring follow-up at 60 months. In the R0-restricted model, surgical margin status was omitted because it was constant within this subgroup. The total retrieved lymph node count was added to the model to determine whether the association between metastatic lymph node count and DFS was independent of the extent of pathological nodal assessment. The 60-month analysis was performed post hoc: DFS events occurring within the first 60 months after surgery were retained, whereas patients whose observed DFS time exceeded 60 months were censored at 60 months, regardless of their subsequent event status.

For exploratory purposes, conventional receiver operating characteristic (ROC) analyses were performed for metastatic lymph node count and primary tumor size, using DFS event status as a binary outcome variable. Optimal cut-off values were determined using the maximum Youden index. Because conventional binary ROC analysis does not account for differences in follow-up time or for censoring, these analyses were regarded as exploratory only and were not used for primary inference. The ROC-derived threshold of 3.5 metastatic lymph nodes was expressed clinically as ≤3 versus ≥4 metastatic lymph nodes and was used only in the exploratory Kaplan–Meier analysis. DFS curves were estimated using the Kaplan–Meier method, and groups were compared using the log-rank test. Numbers at risk were displayed beneath the survival curves.

## 3. Results

### 3.1. Patient Selection and Baseline Characteristics

**Figure 1:**
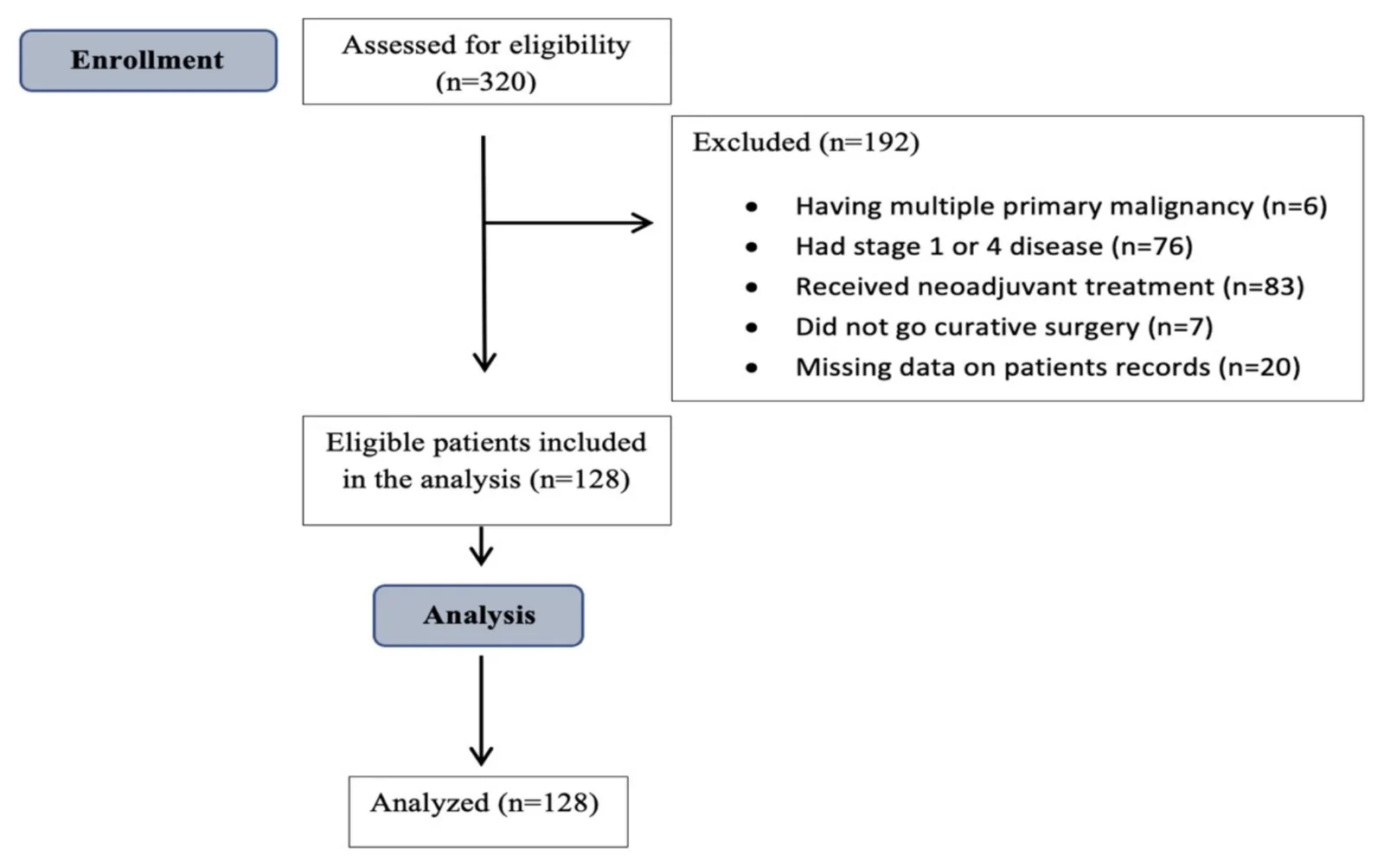
Flow chart of included patients.

A total of 320 patient records were assessed for eligibility. Of these, 192 were excluded because of multiple primary malignancies (*n* = 6), pathological stage I or IV disease (*n* = 76), receipt of neoadjuvant treatment (*n* = 83), surgery not performed with curative intent (*n* = 7), or insufficient clinical or pathological information in the medical records (*n* = 20). The remaining 128 patients were included in the final analysis (Figure 1).

The median age was 62 years (range, 43–87 years), and 45 patients (35.2%) were women. Surgical margins were microscopically negative in 119 patients (93.0%), and 82 patients (64.1%) underwent D2 lymph node dissection. The median total retrieved lymph node count was 20.5 (range, 3–74), and the median metastatic lymph node count was 3.5 (range, 0–64). Pathological stage III disease was present in 69 patients (53.9%). Adjuvant chemotherapy was administered to 95 patients (74.2%) and adjuvant chemoradiotherapy to 74 patients (57.8%). The distribution of pT category was pT2 in 16 patients (12.5%), pT3 in 79 (61.7%), and pT4 in 33 (25.8%); the corresponding pN distribution was pN0 in 29 (22.7%), pN1 in 29 (22.7%), pN2 in 28 (21.9%), and pN3 in 42 (32.8%). Detailed baseline characteristics are presented in Table 1.

### 3.2. Clinicopathological Characteristics According to Surgery Period

Surgery was performed before 2012 in 63 patients and in 2012 or later in 65 patients. In the later period, pT4 disease (38.5% vs. 12.7%; *p* = 0.004), pathological stage III disease (66.2% vs. 41.3%; *p* = 0.005), and D2 lymph node dissection (73.8% vs. 54.0%; *p* = 0.019) were all more frequent. The median number of retrieved lymph nodes was also higher in the later period [24 (range, 3–74) vs. 18 (range, 3–64); *p* = 0.012]. Age, smoking history, tumor size, pN category, and metastatic lymph node count did not differ significantly between the two periods (Supplementary Table S1).

### 3.3. DFS Outcomes and Primary Cox Regression Analysis

A total of 86 DFS events were observed during follow-up: 82 documented recurrences and 4 deaths without previously documented recurrence. Forty-two patients were censored. The median follow-up estimated by the reverse Kaplan–Meier method was 114.3 months (95% CI, 104.6–124.1 months) for the entire cohort. The median follow-up was 155.7 months (95% CI, 106.8–204.6 months) in patients who underwent surgery before 2012 and 58.8 months (95% CI, 54.2–63.4 months) in those who underwent surgery in 2012 or later, and the distributions of follow-up time differed significantly between the periods (log-rank χ^2^ = 59.334; *p* < 0.001). This difference reflected the different potential follow-up times between each patient’s date of surgery and the common database closure date. The median DFS was 40.8 months (95% CI, 20.6–61.1 months).

Among the 82 patients with documented recurrence, the most frequent sites of first recurrence were the liver (*n* = 23; 28.0%), the peritoneum (*n* = 16; 19.5%), and the lung (*n* = 16; 19.5%). These were followed by locoregional recurrence (*n* = 8; 9.8%), distant lymph nodes (*n* = 3; 3.7%), bone (*n* = 2; 2.4%), the ovary (*n* = 1; 1.2%), and the bladder (*n* = 1; 1.2%); the remaining 12 patients (14.6%) had synchronous multisite recurrence.

In the primary multivariable Cox regression model, each additional metastatic lymph node was associated with an approximately 4.9% increase in the hazard of a DFS event (HR, 1.049; 95% CI, 1.022–1.076; *p* < 0.001). Surgery performed in 2012 or later was also associated with a higher hazard of a DFS event compared with surgery before 2012 (HR, 2.104; 95% CI, 1.254–3.531; *p* = 0.005). Smoking history, surgical margin status, tumor size, pT category, ECOG performance status, and adjuvant chemotherapy did not reach statistical significance in the multivariable analysis (Table 2).

The addition of the Box–Tidwell transformation term did not significantly improve model fit (likelihood-ratio χ^2^ = 1.489; df = 1; *p* = 0.222), nor was the transformation term itself statistically significant (*p* = 0.275). These results were consistent with a linear association between metastatic lymph node count and the log hazard.

### 3.4. Alternative Nodal Metrics and Sensitivity Analyses

All four nodal metrics—metastatic lymph node count, pN category, LNR, and LODDS—significantly improved model fit when added separately to the base clinical model. The LNR model yielded the lowest AIC (690.6), followed by LODDS (693.2), compared with 701.2 for both the metastatic lymph node count and pN category models. These within-cohort differences in relative model fit were not interpreted as evidence of clinical superiority of any single nodal metric (Table 3).

The association between metastatic lymph node count and DFS persisted in the model stratified by pT category, in the analysis restricted to patients with R0 resection, in the model additionally adjusted for the total retrieved lymph node count, and in the analysis with follow-up restricted to 60 months. In the *post hoc* sensitivity analysis with follow-up restricted to 60 months, 45 patients were administratively censored at 60 months, and 69 DFS events were included in the analysis. Across these analyses, the adjusted HRs ranged from 1.043 to 1.052, and all associations remained statistically significant (all *p* ≤ 0.007; Table 4).

In the primary Cox model, there was no evidence of violation of the proportional hazards assumption for metastatic lymph node count, surgery period, or ECOG performance status (Supplementary Table S2). Although the global test for pT category was not significant (*p* = 0.077), the pT4-by-log-time interaction was significant (*p* = 0.024), suggesting a possible time-dependent effect. In the sensitivity analysis stratified by pT category, the estimate for metastatic lymph node count was unchanged.

After the total retrieved lymph node count was additionally included in the model, the association between metastatic lymph node count and DFS persisted (HR, 1.043; 95% CI, 1.011–1.075; *p* = 0.007). The total retrieved lymph node count itself was not significantly associated with DFS (HR, 1.008; 95% CI, 0.987–1.028; *p* = 0.474). In this model, surgery performed in 2012 or later also remained associated with a higher hazard of a DFS event (HR, 2.001; 95% CI, 1.170–3.422; *p* = 0.011).

### 3.5. Exploratory ROC and Kaplan–Meier Analyses

**Figure 2:**
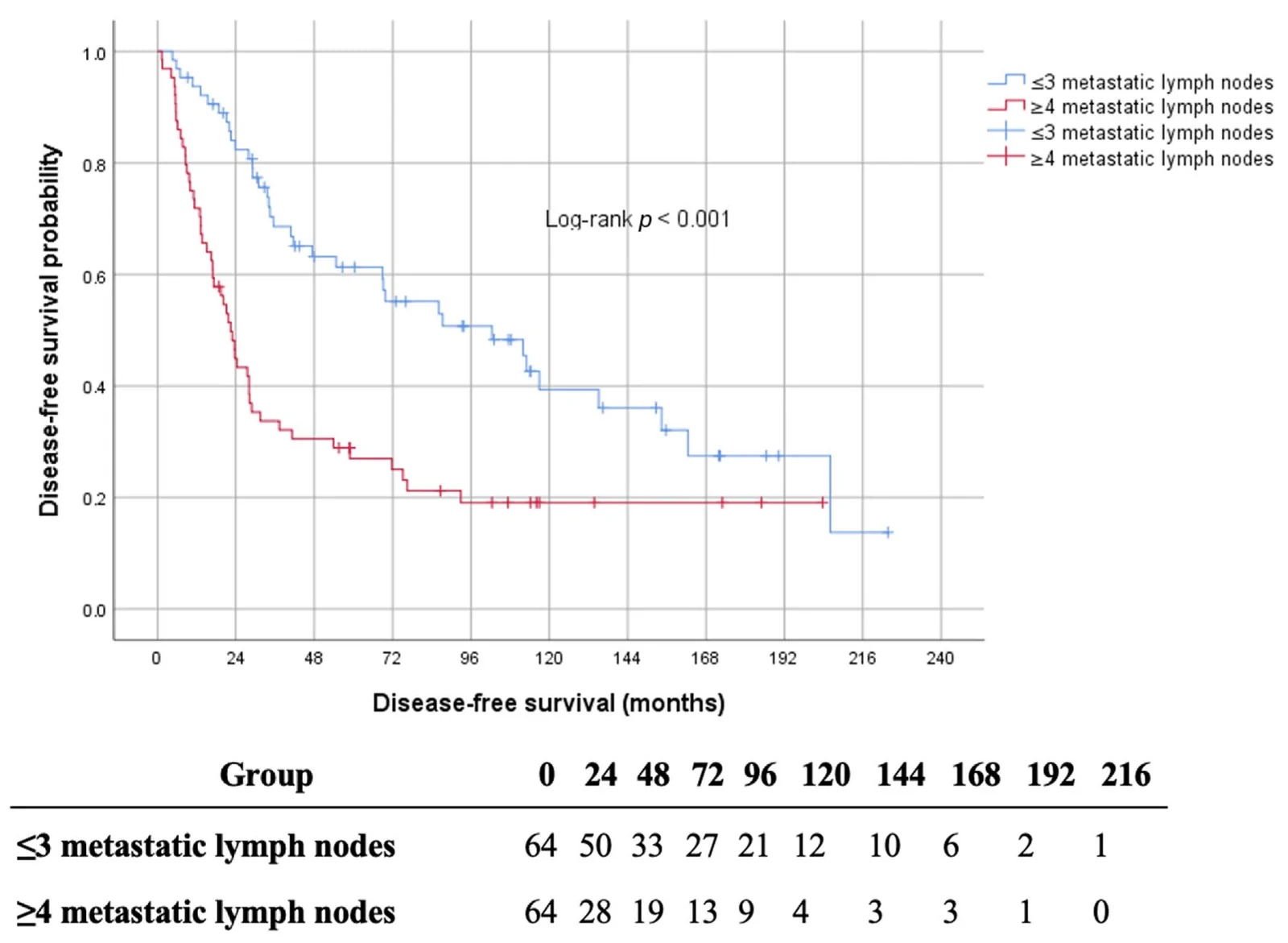
Kaplan–Meier curves for disease-free survival according to metastatic lymph node count group (≤3 vs. ≥4 metastatic lymph nodes).

In the exploratory ROC analysis, metastatic lymph node count showed modest discrimination for DFS event status (AUC, 0.656; 95% CI, 0.557–0.754; *p* = 0.004). The Youden-derived cut-off of 3.5 was expressed clinically as ≤3 vs. ≥4 metastatic lymph nodes. Primary tumor size showed no discriminatory ability (AUC, 0.490; *p* = 0.857; Supplementary Table S3). In the exploratory Kaplan–Meier analysis, the median DFS was 102.4 months (95% CI, 55.6–149.2 months) in patients with ≤3 metastatic lymph nodes and 22.4 months (95% CI, 17.7–27.1 months) in those with ≥4 metastatic lymph nodes. The difference between the groups was statistically significant (log-rank χ^2^ = 15.477; *p* < 0.001; Figure 2 and Supplementary Table S4). Because this grouping was derived from a conventional ROC analysis that did not account for censoring, it was used only for exploratory visualization.

## 4. Discussion

In this retrospective cohort study, metastatic lymph node count was independently associated with DFS in patients with pathological stage II–III GC treated with a surgery-first approach. In the primary multivariable model, each additional metastatic lymph node corresponded to an approximately 4.9% higher hazard of a DFS event, and this association persisted across all four sensitivity analyses. In contrast, primary tumor size and smoking history were not independently associated with DFS in the multivariable model. Surgery performed in 2012 or later was also associated with a higher hazard of a DFS event, although this temporal association should not be interpreted as causal.

Regional lymph node involvement is one of the principal determinants of postoperative prognosis in GC. Previous studies have reported that a higher LNR is associated with early recurrence and shorter survival in patients with pathological stage II–III disease [9,10]. Similarly, LNR has been shown to provide prognostic information for recurrence and survival after curative-intent gastrectomy [12], as well as in broader surgical cohorts including elderly patients and patients with more heterogeneous stage distributions [13,14]. The pT and pN categories have also been reported to be associated with both DFS and OS in stage I–III GC [15]. Consistent with these reports, the association of metastatic lymph node count with DFS in the present cohort persisted after adjustment for pT category, surgical margin status, ECOG performance status, adjuvant chemotherapy, tumor size, and the other prespecified covariates.

Treating metastatic lymph node counts as continuous variables allowed assessment of the graded effect of nodal disease burden, which can be lost when patients are grouped into categories. The Box–Tidwell test provided no evidence of departure from linearity. However, this does not entirely exclude more complex nonlinear relationships. Given that metastatic lymph node count ranged from 0 to 64 with sparse observations at the upper end of the distribution, the sample size may be insufficient to support more flexible nonlinear modeling. Nevertheless, the narrow range of the metastatic lymph node estimate across the sensitivity analyses (adjusted HR, 1.043–1.052) supports the stability of the association within this cohort. All four nodal metrics improved model fit when added separately to the base clinical model, with LNR yielding the lowest AIC and LODDS the second lowest. It has previously been suggested that alternative nodal classification systems may contribute to prognostic stratification beyond the conventional pN category [8]. The discriminatory ability of LODDS has been reported to diminish relative to LNR in series with an adequate number of retrieved lymph nodes [11,20]. However, it has been suggested that this difference may reflect the cut-off values applied rather than LODDS itself, and that with lower thresholds LODDS may remain discriminatory even in patients with adequate nodal retrieval [8]. The median total retrieved lymph node count of 20.5 in the present cohort may be relevant in this context, although these comparisons in the literature are sensitive to the choice of cut-off values, whereas LNR and LODDS were modeled as continuous variables here. Moreover, the nodal metrics were not mutually adjusted but were added separately to the same base clinical model. Therefore, the finding that LNR had the lowest AIC reflects only relative model fit within this cohort and should not be regarded as evidence of superior discrimination, calibration, clinical utility, or external validity.

In the exploratory ROC analysis, metastatic lymph node count showed modest discrimination for DFS event status. Because metastatic lymph node count is an integer variable, the Youden-derived value of 3.5 was expressed clinically as ≤3 vs. ≥4 metastatic lymph nodes. Although the difference in median DFS between these groups was pronounced, conventional binary ROC analysis does not account for follow-up time or censoring. Moreover, the threshold was both derived and evaluated in the same cohort, which may lead to optimistic performance estimates. The threshold of ≥4 metastatic lymph nodes should therefore not be regarded as a validated clinical cut-off or a new staging category, but only as a hypothesis-generating, cohort-specific grouping used for visualization. Primary inference is more appropriately based on the Cox regression analysis, in which metastatic lymph node count was modeled as a continuous variable.

One notable finding of this study was the higher hazard of a DFS event observed in patients who underwent surgery in 2012 or later. The calendar period has previously been shown to be associated with prognosis after gastrectomy in the Swedish Gastric Cancer Surgery Study; in that study, however, more recent surgery was associated with lower mortality [21]. A population-based analysis from the United States similarly reported improved survival in patients resected in more recent periods, accompanied by increased use of radiotherapy and a higher proportion of patients with at least 15 lymph nodes evaluated [22]. A Swedish national registry study also showed that the use of D1+/D2 lymphadenectomy increased over time and was associated with better adjusted survival than more limited dissection [23].

The inverse direction of the association in the present study may therefore reflect differences in patient and disease characteristics between the periods rather than a directly harmful effect of the calendar year of surgery. Patients treated in the more recent period more frequently had pT4 and pathological stage III disease, underwent D2 dissection more often, and had a higher number of retrieved lymph nodes. Referral patterns, preoperative imaging, selection of patients for surgery, pathological examination, stage migration, choice of adjuvant treatment, and the way recurrence was detected may also have changed over the years. In particular, because the frequency and resolution of imaging were more limited in the earlier period, some recurrences may not have been documented. This could have made DFS appear longer in patients treated during that period, and detection bias may account for part of the observed association. Although the calendar period association persisted in the model adjusted for the total retrieved lymph node count and in the analysis with administrative censoring at 60 months, this does not eliminate residual confounding. The finding should therefore not be interpreted as indicating that more recent treatment leads to worse outcomes, but rather as an unexplained association that remains open to period-related confounding.

Smoking history was associated with shorter DFS in univariable analysis but was no longer significant after multivariable adjustment. This association may be sensitive to how the exposure is defined: a smoking duration of more than 30 years has been independently linked to worse survival after gastrectomy [24], and continued smoking after diagnosis has been reported to increase mortality [25]. In the present study, smoking was recorded only as a binary variable, so pack-years, duration, and cessation could not be evaluated. The finding therefore does not indicate that smoking lacks prognostic relevance, but only that this coarse measure showed no independent association with DFS.

The literature on the prognostic role of primary tumor size is heterogeneous. Tsutsuyama et al. reported a tumor size of 5 cm or greater as an adverse prognostic factor in patients with stage II–III disease receiving postoperative S-1 monotherapy [16]. Another study in patients with node-negative disease showed a progressive decline in survival with increasing tumor size [17]. Del Rio et al. identified a lower ROC-derived threshold of 2.5 cm [18], whereas a large cohort including patients with stage I–IV disease and both curative and non-curative resections adopted a 5 cm cut-off, with tumor size remaining independently prognostic across all pathological stages except in T1 tumors [19]. In a different patient group undergoing surgery after neoadjuvant chemotherapy, tumor size was not significantly associated with survival, whereas the number of positive lymph nodes emerged as the strongest prognostic factor [26].

In the present study, primary tumor size was not associated with DFS in either univariable or multivariable Cox regression and showed no discriminatory ability in the exploratory ROC analysis. This finding should not, however, be interpreted as indicating that tumor size lacks prognostic value in GC in general. Because the pT category reflects the depth of tumor invasion and the metastatic lymph node count reflects the burden of regional spread, including these variables in the same model may have attenuated part of the prognostic information that tumor size could otherwise provide. Consistent with this, the association of tumor size with survival has been reported to be partly explained by its close relationship with depth of invasion and lymph node metastasis [18]. Tumor size has been shown to carry prognostic value independent of T stage in cohorts without nodal metastasis [17], whereas tumor size measures may lose their independent contribution in multivariable models that include pT and pN categories [15]. The observation that the independent prognostic value of tumor size was demonstrated in patients receiving adjuvant S-1 but not in the surgery-only group [16] suggests that its prognostic relevance may vary according to the adjuvant treatment approach. The fact that this series also modeled nodal burden categorically rather than as a continuous variable [19] may have contributed to the difference from the present findings. The effect of tumor size may also differ across specific pT groups, nodal subgroups, Lauren subtypes, or anatomical locations, and the present sample size is not sufficient to evaluate such interaction and subgroup analyses reliably. A structured comparison of studies evaluating nodal metrics and primary tumor size, including the present study, is provided in Supplementary Table S5.

Adjuvant chemotherapy also did not reach statistical significance in the primary multivariable model. This finding should not, however, be taken to indicate that adjuvant treatment provides no clinical benefit. Receipt of treatment is a postoperative variable that may be influenced by postoperative recovery, performance status, pathological stage, comorbidities, surgical complications, and physician preference. Consistent with this, in a nationwide population-based cohort, the survival benefit of preoperative chemotherapy observed in the unadjusted analysis (HR, 0.72; 95% CI, 0.62–0.82) did not persist after adjustment for other prognostic factors (HR, 0.92; 95% CI, 0.78–1.08); the authors emphasized that this may reflect unmeasured confounding and should not change clinical practice [21]. In addition, the regimens used changed over the study period, and details concerning the timing of treatment initiation, dose intensity, dose reductions, interruptions, and completion of the planned treatment were not consistently recorded for all patients. This study was therefore not designed to compare the efficacy of adjuvant treatment and should not be interpreted as such.

### Study Limitations

This study has several limitations. First, the retrospective, single-center design is susceptible to bias in patient selection, data recording, and detection of recurrence. Because the institutional records span a long period and the eligible surgical cohort was treated between March 2003 and October 2019, surgical techniques, approaches to lymphadenectomy, imaging modalities, pathological examination, staging, adjuvant treatment, and follow-up practices changed over time. Follow-up was also inherently shorter in the more recent surgery period, which complicates the interpretation of comparisons between periods.

Second, although excluding patients who received neoadjuvant treatment increased the clinical homogeneity of the surgery-first cohort, it also limits the generalizability of the results to patients with locally advanced GC who are treated with perioperative therapy today. Third, recording smoking history only as a binary variable may have led to exposure misclassification. Fourth, incomplete details on the timing, dose intensity, reductions, and interruptions of adjuvant treatment, as well as on its completion, precluded a comprehensive assessment of treatment effects.

Fifth, the composite DFS endpoint included recurrence or death from any cause. Because the causes of death in the four patients who died without documented recurrence could not be reliably determined, an additional sensitivity analysis based on cause of death was not feasible. Sixth, although 128 patients and 86 DFS events provided a reasonable number of events for the primary model, this sample was limited for subgroup analyses, interaction testing, flexible nonlinear modeling, and internal validation. The proportional hazards assumption was formally assessed for the primary model, whereas the alternative nodal models were treated as exploratory comparisons of model fit and were not subjected to separate formal time-interaction analyses.

Finally, the comparison of alternative nodal models relies on AIC values within the same cohort and does not include optimism-corrected internal validation or external validation. The exploratory ROC analysis does not account for censoring, and because the threshold was derived in the same dataset, it carries a risk of optimism. The relative performance of the nodal metrics and the threshold of ≥4 metastatic lymph nodes should therefore not be regarded as validated clinical tools.

The strengths of this study include complete data for all covariates in the primary Cox model, retrospective restaging of all cases according to the eighth edition of the AJCC/UICC classification, detailed verification of DFS events as either recurrence or death without recurrence, estimation of the median follow-up using the reverse Kaplan–Meier method, and specification of the primary model with clinically prespecified variables rather than automated variable selection. In addition, overlapping nodal metrics were not used simultaneously in the same model, and the association of metastatic lymph node count with DFS persisted across multiple sensitivity analyses, supporting the robustness of the main finding within this cohort.

## 5. Conclusions

In conclusion, in this retrospective single-center cohort of patients with pathological stage II–III GC treated with a surgery-first approach, a higher metastatic lymph node count was independently associated with shorter DFS, whereas primary tumor size was not independently associated with DFS after adjustment for prespecified clinical and pathological factors. LNR, LODDS, pN category, and metastatic lymph node count each improved relative model fit when evaluated separately, and LNR yielded the lowest AIC in this cohort; these analyses, however, do not establish clinical superiority or external validity. The exploratory threshold of four metastatic lymph nodes should not be regarded as a validated clinical cut-off, and the observed association with calendar period should be interpreted cautiously in view of possible residual confounding. External validation in larger, multicenter cohorts is warranted before these findings can inform clinical decision-making.

## Figures and Tables

**Table 1:** Baseline clinicopathological and treatment characteristics of the study population (*n* = 128).

Characteristic	Value
**Age,** years	62 (43–87)
**Sex**	
Female	45 (35.2)
**Comorbidity**	
Yes	59 (46.1)
**Smoking history**	
Yes	55 (43.0)
**Surgery period**	
Before 2012	63 (49.2)
2012 or later	65 (50.8)
**Surgical margin**	
Negative	119 (93.0)
Positive	9 (7.0)
**Gastrectomy type**	
Total	59 (46.1)
Subtotal	69 (53.9)
**Lymph node dissection**	
D1	46 (35.9)
D2	82 (64.1)
**Retrieved lymph nodes, median**	20.5 (3–74)
**Metastatic lymph nodes, median (range)**	3.5 (0–64)
**Metastatic lymph node category**	
≤3	64 (50.0)
≥4	64 (50.0)
**Tumor size, centimeter**	6.0 (0.6–14.0)
LODDS	−1.536 (−4.344 to 4.860)
**Lauren classification**	
Intestinal	100 (78.1)
Diffuse	20 (15.6)
Mixed	2 (1.6)
Other	6 (4.7)
**Tumor location**	
Cardia	33 (25.8)
Corpus	29 (22.7)
Antrum/pylorus	61 (47.7)
Linitis plastica	5 (3.9)
**Histological type**	
Signet-ring cell carcinoma	15 (11.7)
Non-signet-ring histology	113 (88.3)
**Histological grade**	
Grade 1	15 (11.7)
Grade 2	80 (62.5)
Grade 3	33 (25.8)
**Pathological T category**	
pT2	16 (12.5)
pT3	79 (61.7)
pT4	33 (25.8)
**Pathological N category**	
pN0	29 (22.7)
pN1	29 (22.7)
pN2	28 (21.9)
pN3	42 (32.8)
**Pathological stage**	
Stage II	59 (46.1)
Stage III	69 (53.9)
**Perineural invasion**	
Present	75 (58.6)
**Vascular invasion**	
Present	81 (63.3)
**Serosal involvement**	
Present	100 (78.1)
**HER2 status** (*n* = 82)	
Positive	16 (19.5)
**ECOG performance status**	
0	93 (72.7)
1	26 (20.3)
2	9 (7.0)
**Adjuvant chemotherapy**	
Yes	95 (74.2)
**Adjuvant chemotherapy regimen** (*n* = 95)	
FUFA/capecitabine	61 (64.2)
CAPOX	19 (20.0)
DCF	7 (7.4)
FOLFOX	4 (4.2)
Other	4 (4.2)
**Adjuvant chemoradiotherapy**	
Yes	74 (57.8)
**DFS status**	
Censored	42 (32.8)
Event	86 (67.2)

Data are presented as n (%) or median (range), unless otherwise indicated. CAPOX, capecitabine plus oxaliplatin; DCF, docetaxel, cisplatin, and fluorouracil; DFS, disease-free survival; ECOG, Eastern Cooperative Oncology Group; FOLFOX, fluorouracil, leucovorin, and oxaliplatin; FUFA, fluorouracil plus folinic acid; HER2, human epidermal growth factor receptor 2; LODDS, log odds of positive lymph nodes.

**Table 2:** Univariable and primary multivariable Cox regression analyses for DFS.

Variable	Comparison/Unit	Unadjusted HR (95% CI)	*p* Value	Adjusted HR (95% CI)	*p* Value
Age	Per 1-year increase	0.991 (0.968–1.015)	0.476	0.987 (0.964–1.010)	0.274
Sex	Male vs. female	1.716 (1.074–2.744)	*0.024*	—	—
Comorbidity	Yes vs. no	1.668 (1.082–2.571)	*0.021*	—	—
Smoking history	Yes vs. no	1.722 (1.118–2.653)	*0.014*	1.411 (0.874–2.278)	0.159
Surgery period	2012 or later vs. before 2012	2.336 (1.463–3.731)	*<0.001*	2.104 (1.254–3.531)	0.005
Positive surgical margin	Yes vs. no	3.013 (1.423–6.382)	*0.004*	2.112 (0.894–4.989)	0.088
Gastrectomy type	Subtotal vs. total	0.612 (0.398–0.940)	*0.025*	—	—
Lymph node dissection	D2 vs. D1	1.550 (0.978–2.457)	0.062	—	—
Tumor location, overall	Cardia as reference	—	0.062	—	—
	Corpus vs. cardia	0.915 (0.491–1.706)	0.780	—	—
	Antrum/pylorus vs. cardia	0.799 (0.477–1.340)	0.395	—	—
	Linitis plastica vs. cardia	2.965 (1.105–7.953)	*0.031*	—	—
Lauren classification, overall	Intestinal as reference	—	0.266	—	—
	Diffuse vs. intestinal	1.632 (0.943–2.826)	0.080	—	—
	Mixed vs. intestinal	1.089 (0.150–7.913)	0.933	—	—
	Other vs. intestinal	0.557 (0.136–2.274)	0.415	—	—
Histological type	Non-signet-ring vs. signet-ring	0.577 (0.318–1.044)	0.069	—	—
Histological grade	Grade 3 vs. Grade 1–2	1.539 (0.970–2.443)	0.067	—	—
Tumor size	Per 1 cm increase	1.020 (0.946–1.099)	0.607	0.950 (0.876–1.030)	0.213
Retrieved lymph nodes	Per 1-node increase	1.023 (1.008–1.039)	*0.003*	—	—
Metastatic lymph node count	Per 1-node increase	1.049 (1.028–1.071)	*<0.001*	1.049 (1.022–1.076)	<0.001
Metastatic lymph node category	≥4 vs. ≤3	2.342 (1.514–3.623)	*<0.001*	—	—
Pathological T category, overall	pT2 as reference	—	*<0.001*	—	0.268
	pT3 vs. pT2	1.587 (0.747–3.373)	0.230	1.792 (0.823–3.904)	0.142
	pT4 vs. pT2	3.672 (1.631–8.270)	*0.002*	2.068 (0.832–5.139)	0.118
Pathological N category, overall	pN0 as reference	—	*<0.001*	—	—
	pN1 vs. pN0	1.080 (0.539–2.165)	0.829	—	—
	pN2 vs. pN0	1.936 (0.979–3.829)	0.058	—	—
	pN3 vs. pN0	3.230 (1.742–5.987)	*<0.001*	—	—
Pathological stage	Stage III vs. II	2.675 (1.699–4.210)	*<0.001*	—	—
ECOG performance status, overall	ECOG 0 as reference	—	*0.015*	—	0.108
	ECOG 1 vs. 0	1.205 (0.718–2.023)	0.480	1.465 (0.843–2.545)	0.176
	ECOG 2 vs. 0	3.278 (1.467–7.328)	*0.004*	2.424 (0.918–6.400)	0.074
Perineural invasion	Present vs. absent	2.028 (1.293–3.180)	*0.002*	—	—
Vascular invasion	Present vs. absent	3.033 (1.824–5.042)	*<0.001*	—	—
Serosal involvement	Present vs. absent	2.064 (1.119–3.806)	*0.020*	—	—
Adjuvant chemotherapy	Yes vs. no	0.745 (0.467–1.190)	0.218	0.592 (0.346–1.014)	0.056
Adjuvant chemoradiotherapy	Yes vs. no	0.919 (0.598–1.414)	0.702	—	—
Adjuvant regimen, overall	No chemotherapy as reference	—	0.102	—	—
	FUFA/capecitabine vs. none	0.655 (0.395–1.088)	0.102	—	—
	CAPOX vs. none	1.070 (0.530–2.161)	0.849	—	—
	DCF vs. none	0.800 (0.306–2.094)	0.650	—	—
	FOLFOX vs. none	0.286 (0.039–2.119)	0.221	—	—
	Other regimen vs. none	2.499 (0.852–7.332)	0.095	—	—
Adjuvant chemotherapy cycles	Per cycle; treated patients only (*n* = 95)	0.840 (0.672–1.052)	0.129	—	—
LNR	Per 0.10 increase	1.225 (1.133–1.324)	*<0.001*	—	—
LODDS	Per 1-unit increase	1.352 (1.186–1.541)	*<0.001*	—	—

The primary multivariable model included age, smoking history, surgery period, surgical margin status, pathological T category, ECOG performance status, adjuvant chemotherapy, tumor size, and metastatic lymph node count. *n* = 128 and 86 DFS events. pN category, positive lymph node ratio, LODDS, stage, and the dichotomized metastatic lymph node variable were not entered simultaneously with metastatic lymph node count because of conceptual overlap and collinearity. CI, confidence interval; DFS, disease-free survival; ECOG, Eastern Cooperative Oncology Group; HR, hazard ratio; LODDS, log odds of positive lymph nodes; LNR, lymph node ratio. The linearity assumption for metastatic lymph node count was assessed using a Box–Tidwell transformation, with no statistically significant evidence of departure from linearity (likelihood-ratio *p* = 0.222).

**Table 3:** Comparison of alternative nodal metrics added separately to the same base clinical Cox model.

Model	Nodal Metric	Adjusted Effect (95% CI)	*p* Value	−2 log Likelihood	Parameters	AIC	LR χ^2^ (df) vs. Base	*p* Value
Base clinical model	None	—	—	689.295	10	709.295	—	—
Model A	Metastatic LN count, per 1 node	1.049 (1.022–1.076)	*<0.001*	679.153	11	701.153	10.142 (1)	*0.001*
Model B	pN category, overall	Overall *p* = 0.003	*0.003*	675.225	13	701.225	14.070 (3)	*0.003*
Model C	LNR, per 0.10 increase	1.243 (1.134–1.363)	*<0.001*	668.632	11	690.632	20.663 (1)	*<0.001*
Model D	LODDS, per 1-unit increase	1.401 (1.203–1.632)	*<0.001*	671.199	11	693.199	18.096 (1)	*<0.001*

The base clinical model included age, smoking history, surgery period, surgical margin status, pathological T category, ECOG performance status, adjuvant chemotherapy, and tumor size. Each nodal metric was added in a separate model. Lower AIC indicates better relative model fit within this cohort; these analyses do not establish external clinical utility or superiority. AIC, Akaike information criterion; CI, confidence interval; df, degrees of freedom; LN, lymph node; LNR, lymph node ratio; LODDS, log odds of positive lymph nodes; LR, likelihood ratio.

**Table 4:** Sensitivity analyses for the association between metastatic lymph node count and DFS.

Analysis	Population/Model Modification	N/DFS Events	Adjusted HR per Metastatic LN (95% CI)	*p* Value
Primary model	All patients; clinically prespecified adjustment	128/86	1.049 (1.022–1.076)	*<0.001*
pT-stratified model	Stratified by pT2/pT3/pT4	128/86	1.049 (1.023–1.077)	*<0.001*
R0-only analysis	Patients with positive margins excluded	119/78	1.046 (1.018–1.075)	*0.001*
Retrieved LN adjusted model	Total retrieved LN count added	128/86	1.043 (1.011–1.075)	*0.007*
60-month restricted analysis	Administrative censoring at 60 months	128/69	1.052 (1.025–1.080)	*<0.001*

All models retained the same clinically prespecified adjustment set, except that surgical margin status was omitted from the R0-only model because it was constant. In the retrieved LN adjusted model, retrieved LN count was not significant (HR 1.008, 95% CI 0.987–1.028; *p* = 0.474). In the 60-month restricted analysis, surgery in 2012 or later also remained associated with a higher DFS-event hazard (adjusted HR 1.992, 95% CI 1.149–3.453; *p* = 0.014). CI, confidence interval; DFS, disease-free survival; HR, hazard ratio; LN, lymph node; R0, microscopically margin negative resection.

## Data Availability

The authors complied with institutional policies regarding patient data confidentiality. Data are available from the corresponding author upon reasonable request.

## Supplementary Materials

The following supporting information can be downloaded at https://www.mdpi.com/article/10.3390/medicina62081528/s1. Table S1: Clinicopathological characteristics according to surgery period; Table S2: Proportional hazards assumption assessments for the primary Cox model; Table S3: Exploratory conventional ROC analyses for DFS event status; Table S4: Exploratory Kaplan–Meier estimates of DFS by metastatic lymph-node category; Table S5: Comparison of Studies Evaluating Nodal Metrics and Primary Tumor Size in Gastric Cancer.
