# Supplementary material for: Association of Metastatic Lymph Node Count and Primary Tumor Size with Disease-Free Survival in Stage II–III Gastric Cancer: A Retrospective Cohort Study
Source: Medicina (Kaunas). 2026 Aug 8;62(8):1528. doi: 10.3390/medicina62081528 (PMC13514888; doi:10.3390/medicina62081528)
Supplement: Supplementary file 1 [file medicina-62-01528-s001.zip › S1 supplementary table S1.pdf]

Supplementary Table S1. Clinicopathological characteristics according to surgery period

Characteristic	Before 2012 (n = 63)	2012 or later (n = 65)	p value
**Age, years**	61 (43–85)	65 (44–87)	0.340
**Smoking history,** yes	24 (38.1)	31 (47.7)	0.273
**Positive surgical margin**	2 (3.2)	7 (10.8)	0.165*
**Primary tumor size,** centimeter	6.0 (0.6–13.0)	6.0 (3.0–14.0)	0.992
**Pathological T category**			***0.004***
pT2	10 (15.9)	6 (9.2)	
pT3	45 (71.4)	34 (52.3)	
pT4	8 (12.7)	25 (38.5)	
**Pathological N category**			0.255
pN0	16 (25.4)	13 (20.0)	
pN1	18 (28.6)	11 (16.9)	
pN2	12 (19.0)	16 (24.6)	
pN3	17 (27.0)	25 (38.5)	
**Pathological stage III**	26 (41.3)	43 (66.2)	***0.005***
**ECOG performance status**			0.185
ECOG 0	46 (73.0)	47 (72.3)	
ECOG 1	15 (23.8)	11 (16.9)	
ECOG 2	2 (3.2)	7 (10.8)	
**D2 lymph-node dissection**	34 (54.0)	48 (73.8)	***0.019***
**Retrieved lymph nodes**	18 (3–64)	24 (3–74)	***0.012***
**Metastatic lymph nodes**	2 (0–64)	4 (0–29)	0.117
**Adjuvant chemotherapy,** yes	42 (66.7)	53 (81.5)	0.054
**Adjuvant chemoradiotherapy,** yes	34 (54.0)	40 (61.5)	0.386

Data are presented as n (%) or median (range). Continuous variables were compared using the Mann–Whitney U test; categorical variables were compared using Pearson's chi-square test. *Two-sided Fisher's exact test was used because of small expected cell counts. ECOG, Eastern Cooperative Oncology Group.
