# Supplementary material for: Association of Metastatic Lymph Node Count and Primary Tumor Size with Disease-Free Survival in Stage II–III Gastric Cancer: A Retrospective Cohort Study
Source: Medicina (Kaunas). 2026 Aug 8;62(8):1528. doi: 10.3390/medicina62081528 (PMC13514888; doi:10.3390/medicina62081528)
Supplement: Supplementary file 1 [file medicina-62-01528-s001.zip › S2 supplementary table S2.pdf]

Supplementary Table S2. Proportional hazards assumption assessments for the primary Cox model

Assessment	*p* value	Interpretation
Metastatic lymph-node count × ln(time)	0.526	No evidence of violation
Surgery period × ln(time)	0.223	No evidence of violation
Age, smoking, margin, adjuvant chemotherapy, and tumor size: global test	0.499	No evidence of violation
ECOG time interactions: global test	0.428	No evidence of violation
Pathological T category time interactions: global test	0.077	Global test not significant
pT4 × ln(time)	0.024	Possible time-dependent effect; addressed by pT-stratified sensitivity model

Time-dependent covariates were generated by interaction with the logarithm of analysis time. The primary metastatic lymph-node estimate was unchanged in the pT-stratified sensitivity analysis.
