# Supplementary material for: Association of Metastatic Lymph Node Count and Primary Tumor Size with Disease-Free Survival in Stage II–III Gastric Cancer: A Retrospective Cohort Study
Source: Medicina (Kaunas). 2026 Aug 8;62(8):1528. doi: 10.3390/medicina62081528 (PMC13514888; doi:10.3390/medicina62081528)
Supplement: Supplementary file 1 [file medicina-62-01528-s001.zip › S3 supplementary table S3.pdf]

Supplementary Table S3. Exploratory conventional ROC analyses for DFS event status

Marker	Outcome	AUC (95% CI)	p value	Youden cut-off	Clinical category	Sensitivity (%)	Specificity (%)
Metastatic lymph node count	DFS event status	0.656 (0.557–0.754)	***0.004***	3.5	≥4	58.1	66.7
Primary tumor size (cm)	DFS event status	0.490 (0.384–0.597)	0.857	9.75	≥9.75 cm	17.4	88.1

The positive state was a DFS event (86 events and 42 censored observations). This conventional binary ROC analysis does not account for censoring and is therefore exploratory; primary inference is based on continuous-variable Cox regression. The Youden cut-off for tumor size is a mathematical data-derived value only; because tumor size showed no discriminatory ability (AUC 0.490; p = 0.857), no clinically useful tumor size threshold was identified. Tied values were present between the event and censored groups. AUC, area under the curve; CI, confidence interval; DFS, disease-free survival; ROC, receiver operating characteristic.
