# Supplementary material for: Association of Metastatic Lymph Node Count and Primary Tumor Size with Disease-Free Survival in Stage II–III Gastric Cancer: A Retrospective Cohort Study
Source: Medicina (Kaunas). 2026 Aug 8;62(8):1528. doi: 10.3390/medicina62081528 (PMC13514888; doi:10.3390/medicina62081528)
Supplement: Supplementary file 1 [file medicina-62-01528-s001.zip › S4 supplementary table S4.pdf]

Supplementary Table S4. Exploratory Kaplan–Meier estimates of DFS by metastatic lymph-node category

Group	N	DFS events	Median DFS, months (95% CI)	Comparison
≤3 metastatic LNs	64	36	102.4 (55.6–149.2)	
≥4 metastatic LNs	64	50	22.4 (17.730–27.137)	
Overall	128	86	40.8 (20.597–61.070)	Log-rank χ^2 = 15.477; $p < 0.001$

The ≤3 versus ≥4 grouping was derived from an exploratory DFS-event ROC analysis. It should be used for visualization only, not as the primary prognostic model. CI, confidence interval; DFS, disease-free survival; LN, lymph node.
