# Supplementary material for: Association of Metastatic Lymph Node Count and Primary Tumor Size with Disease-Free Survival in Stage II–III Gastric Cancer: A Retrospective Cohort Study
Source: Medicina (Kaunas). 2026 Aug 8;62(8):1528. doi: 10.3390/medicina62081528 (PMC13514888; doi:10.3390/medicina62081528)
Supplement: Supplementary file 1 [file medicina-62-01528-s001.zip › S5 upplementary Table S5.pdf]

Supplementary Table S5. Comparison of Studies Evaluating Nodal Metrics and Primary Tumor Size in Gastric Cancer

Part A. Studies Evaluating Nodal Metrics

Study (year)	Design and population	Stage and treatment	Prognostic variable	Cut-off or categorization	Adjustment variables	Endpoint	Main finding	Incremental value beyond standard staging
**Kano et al. (2020)**	Retrospective, single-center study including 838 patients who underwent microscopically curative gastrectomy with at least 16 harvested lymph nodes between 2000 and 2018.	Pathological stage II-III gastric adenocarcinoma. Preoperative chemotherapy or radiotherapy was excluded; adjuvant treatment was generally administered according to stage and contemporary practice.	LNR compared with pathological N stage.	X-tile-derived LNR groups: 0-0.10, >0.10-0.25, and >0.25.	RFS model: macroscopic type, differentiation, serosal invasion, LNR, lymphatic invasion, and vascular invasion. OS model additionally included age. pN was not entered simultaneously with LNR.	RFS and OS.	Intermediate and high LNR independently predicted worse RFS and OS. Adjusted HRs were 3.128 and 5.148 for RFS, and 2.749 and 4.654 for OS, compared with low LNR.	Supported, particularly in stage III. Time-dependent ROC analyses suggested better discrimination than pN in stage III, but formal incremental value was not tested in a combined model.
**Supsamutchai et al. (2020)**	Retrospective, single-center cohort of 153 patients undergoing curative-intent gastric resection from 1995 to 2016.	Gastrectomy with D2 lymphadenectomy. Neoadjuvant therapy was excluded; postoperative adjuvant therapy was given according to institutional practice.	LNR, retrieved lymph node count, and absolute metastatic lymph node count.	Primary LNR categories: 0-0.35, >0.35-0.75, >0.75-1.00. Multivariable analysis used <=0.35 versus >0.35. Secondary ROC suggested 0.25.	Variables with p<0.10 in univariable analysis. Model included pN, metastatic LN count, retrieved LN count (<15 vs >=15), LNR, and TNM stage (I-II vs III).	RFS/local recurrence.	LNR >0.35 independently predicted recurrence (adjusted HR 8.53, 95% CI 1.97-36.86). pN, metastatic LN count, retrieved LN count, and TNM stage were not significant after adjustment.	Supported after adjustment for conventional nodal and TNM variables, but limited by only 28 recurrence events, a wide confidence interval, and no external validation.
**Miciak et al. (2025)**	Retrospective, single-center study of 194 patients operated on between 2017 and 2021. Main LNR analysis included 133 patients undergoing gastrectomy with D2 lymphadenectomy; resection was abandoned in 61 patients.	Heterogeneous stages 0-IV. Perioperative chemotherapy was administered to 51.5%; FLOT, EOX, and ECX were used.	LNR.	No validated or survival-derived cut-off. Mean LNR was 0.134 and was used descriptively as <=0.134 versus >0.134.	None. Analyses consisted mainly of group comparisons and Pearson correlation; no multivariable Cox model adjusting for TNM and other prognostic factors.	Survival time in months and postoperative complications.	LNR increased with higher T, N, M, and overall stage and correlated negatively with survival. Mean survival was 32.5 months for LNR <=0.134 and 19.9 months for LNR >0.134.	Not demonstrated. The study showed unadjusted correlations with stage and survival but did not establish independent or incremental prognostic value beyond TNM.
**Tsai et al. (2025)**	Retrospective, single-center study. Of 604 screened patients, 237 patients aged >=65 years were included.	Pathological stage II-III gastric cancer treated with curative surgery and D1 or D2 lymphadenectomy from 2009 to 2020. Neoadjuvant chemotherapy was excluded; 71.3% received adjuvant chemotherapy.	LNR.	ROC-derived cut-off: <=0.17 versus >0.17; AUC 0.758 for early recurrence.	Multivariable logistic model included BMI, ECOG PS, tumor size, gastrectomy type, pN stage, LNR, lymphovascular and perineural invasion, margin, adjuvant chemotherapy, CEA, and CA19-9.	Early recurrence within 2 years.	LNR >0.17 was the only statistically significant independent predictor of early recurrence (adjusted OR 5.30, 95% CI 2.07-13.53). pN was not significant after adjustment.	Supported beyond pN for early-recurrence prediction, but the threshold was derived and tested in the same dataset and was not internally or externally validated.
**Grigorie et al. (2026)**	Retrospective, single-center pilot study. Seventy-five patients underwent curative-intent gastrectomy with D2 lymphadenectomy; OS analysis included 71 postoperative survivors.	Nonmetastatic gastric adenocarcinoma, stages I-III. Eighteen patients received neoadjuvant chemotherapy; 57 underwent upfront surgery.	LNR and LODDS, compared with pathological N stage.	LNR: <=0.08, >0.08-0.33, >0.33. LODDS: <=-2.2, >-2.2 to -0.6, >-0.6. Data-derived by X-tile.	Variables significant in univariable analysis entered into a backward stepwise Cox model, including tumor grade, N stage, LNR, and LODDS. Interpretation was limited by perfect collinearity between LNR and LODDS categories.	OS. DFS was not analyzed because recurrence data were incomplete.	Consecutive N-stage groups did not adequately separate OS. Higher LNR and LODDS categories were associated with poorer OS and produced clearer survival stratification.	Suggested, but not definitively demonstrated. Findings favored LNR/LODDS over N stage, but the study was limited by small sample size, data-derived thresholds, collinearity, and no external validation.

Part B. Studies Evaluating Primary Tumor Size

Study (year)	Design and population	Stage and treatment	Prognostic variable	Cut-off or categorization	Adjustment variables	Endpoint	Main finding	Incremental value beyond standard staging
**Zu et al. (2013)**	Retrospective, single-center study of 1800 patients undergoing gastrectomy from 1997 to 2007; 1320 underwent curative gastrectomy.	Gastric adenocarcinoma, stages I-IV. No preoperative chemotherapy or radiotherapy. Both curative and non-curative resections were included; adjuvant-treatment data were unavailable.	Maximum pathological tumor diameter.	<=5 cm versus >5 cm. The threshold was selected by evaluating survival at 1-cm intervals and maximizing the chi-square statistic.	Main Cox models included age, tumor size, lymph node metastasis, depth of invasion, and Borrmann type or curability. Additional analyses were stratified by pT, pN, and pTNM.	OS/disease-specific survival reporting.	Tumor size >5 cm independently predicted worse survival in the curative cohort (HR 1.76) and overall cohort (HR 1.72). The association persisted within pT2-4, N0-3, and TNM stages I-IV, but not pT1.	Supported by pT-, pN-, and pTNM-stratified analyses, suggesting complementary information. Formal improvement in model discrimination or reclassification was not assessed.

**Xu et al. (2014)**	Retrospective analysis of a prospectively maintained single-center database including 492 patients with node-negative gastric cancer.	pT1-4aN0M0 disease treated with R0 gastrectomy and at least D2 lymphadenectomy from 1995 to 2008. More than 15 nodes were examined in every patient; no neoadjuvant chemotherapy.	Maximum primary tumor diameter.	ROC-derived cut-off: <4.75 cm versus >=4.75 cm. Tumor size was also evaluated continuously and in 1-cm increments.	Main Cox model included tumor size, depth of invasion, and Borrmann type. Additional models compared T stage alone with T stage plus tumor size; bootstrap resampling provided internal validation.	Five-year OS and prognostic prediction accuracy.	Tumor size and invasion depth independently predicted poor survival. Adding tumor size increased predictive accuracy from 72.2% to 76.4% (+4.2%), with significant gains in pT3N0M0 (+3.9%) and pT4aN0M0 (+6.5%).	Demonstrated in node-negative disease: adding tumor size to T stage significantly improved internally validated prognostic accuracy, particularly in pT3-4a disease.
**Del Rio et al. (2017)**	Retrospective study of patients who underwent surgery for gastric cancer between 1996 and 2010.	Surgically treated gastric cancer. Disease-stage distribution and neoadjuvant or adjuvant treatment details were not reported in the available abstract.	Primary tumor size; associations with depth of invasion and lymph node metastasis were also evaluated.	ROC-derived cut-off: 2.5 cm. Groups were defined as <2.5 cm, 2.5 to <5 cm, and >=5 cm.	No multivariable adjustment strategy was reported in the available abstract.	OS.	Tumor size was significantly associated with depth of invasion and lymph node metastasis. OS was higher in patients with tumors <2.5 cm than in those with tumors >=2.5 cm (p<0.01).	Not demonstrated. The available abstract did not report a multivariable model or formal testing of predictive improvement beyond standard staging.
**Tsutsuyama et al. (2020)**	Retrospective, multi-institutional study including 847 patients who underwent R0 gastrectomy between 2010 and 2014.	Pathological stage II-III gastric cancer without preoperative treatment. Patients underwent surgery alone or postoperative S-1 monotherapy.	Macroscopic primary tumor size.	<50 mm versus >=50 mm. The 50-mm threshold from a previous study was reassessed by ROC analysis.	In the S-1 group, multivariable analysis included CA19-9, tumor size, gastrectomy type, operative time, differentiation, lymphatic and vascular invasion, invasive growth, pT4, pN2-3, and UICC stage III.	OS; RFS and initial recurrence patterns were also assessed.	In the S-1 group, tumor size >=50 mm independently predicted poorer OS (adjusted HR 1.57, 95% CI 1.03-2.41) and more peritoneal and nodal recurrence. It was not independent in the surgery-alone group.	Supported in the adjuvant S-1 subgroup after adjustment for pT, pN, and overall stage. Formal improvement in discrimination beyond TNM was not tested, and the finding was treatment-specific.
**Uzun et al. (2020)**	Retrospective, single-center study of 240 surgically treated patients. Prognostic analysis was restricted to 46 patients receiving neoadjuvant chemotherapy; 194 underwent upfront surgery.	Predominantly stage II-III gastric cancer. Neoadjuvant chemotherapy was followed by gastrectomy and D2 lymphadenectomy. Positive margins, intraoperative metastasis, early postoperative death, and initial T1 disease were excluded.	Primary tumor size analyzed continuously.	No tumor-size cut-off. Median postoperative tumor size in the neoadjuvant group was 4.8 cm (IQR 3.0-7.0).	Cox model included sex, age, location, Borrmann and Lauren classifications, tumor size, invasion depth, pN stage, retrieved LN count, vascular invasion, and perineural invasion.	OS.	Tumor size was not significantly associated with OS after adjustment (effect estimate 1.305, 95% CI 0.981-1.736; p=0.068). pN stage remained the principal independent prognostic factor.	Not demonstrated. Interpretation is limited by the very small neoadjuvant cohort and imprecise estimates.

Part C. Present Study

Study (year)	Design and population	Stage and treatment	Prognostic variable	Cut-off or categorization	Adjustment variables	Endpoint	Main finding	Incremental value beyond standard staging
**Present study (2026)**	Retrospective, single-center cohort of 128 patients (86 DFS events). Institutional records from January 2001 to January 2024 were screened; eligible surgery-first patients underwent gastrectomy between March 2003 and October 2019.	Pathological stage II–III gastric cancer treated with curative-intent gastrectomy using a surgery-first approach; neoadjuvant treatment was excluded, and 95 patients (74.2%) received adjuvant chemotherapy.	Metastatic LN count, pN category, LNR, LODDS, and primary tumor size.	Metastatic LN count, LNR, LODDS, and primary tumor size were modeled as continuous variables; pN was categorical. Exploratory conventional DFS-event ROC analysis yielded a 3.5-node threshold, expressed as ≤3 versus ≥4 metastatic LNs; no clinically useful tumor-size threshold was identified. Primary tumor size showed no discriminatory ability (AUC 0.490).	Base clinical model: age, smoking history, surgery period, margin status, pT category, ECOG PS, adjuvant chemotherapy, and primary tumor size. Metastatic LN count, pN category, LNR, and LODDS were each added separately; the primary model comprised the base clinical model plus metastatic LN count.	DFS only (recurrence or death from any cause, whichever occurred first).	Each additional metastatic LN was associated with a 4.9% higher hazard of a DFS event (adjusted HR 1.049, 95% CI 1.022–1.076; p<0.001), and the association persisted across four sensitivity analyses. Primary tumor size was not independently associated with DFS (adjusted HR per cm 0.950, 95% CI 0.876–1.030; p=0.213). All four nodal metrics improved model fit; LNR yielded the lowest AIC (690.632), followed by LODDS (693.199).	Not formally demonstrated beyond standard pN staging, because the nodal metrics were evaluated in separate models rather than added to a model containing pN. LNR yielded the lowest within-cohort AIC, but external predictive superiority, calibration, reclassification, and clinical utility were not established.

Abbreviations: AUC, area under the curve; BMI, body mass index; CEA, carcinoembryonic antigen; DFS, disease-free survival; ECOG PS, Eastern Cooperative Oncology Group performance status; FLOT, fluorouracil, leucovorin, oxaliplatin, and docetaxel; LN, lymph node; LNR, lymph node ratio; LODDS, log odds of positive lymph nodes; OS, overall survival; pN, pathological nodal stage; pT, pathological tumor stage; RFS, recurrence-free survival; ROC, receiver operating characteristic; TNM, tumor-node-metastasis.

Interpretive note: "Supported" indicates evidence of prognostic association after adjustment for conventional staging variables, whereas "Demonstrated" is reserved for studies that formally tested and showed improved predictive performance beyond a standard staging model.
